# Is amnesia in women victims of sexual assault a risk factor for developing PTSD symptoms?

**DOI:** 10.1080/13218719.2025.2546314

**Published:** 2025-10-06

**Authors:** Camille Jantzi, Thomas Demessence, Gérard Niveau, Jasmine Abdulcadir, Tony Fracasso

**Affiliations:** a Unit of Forensic Psychiatry, University Centre of Legal Medicine Lausanne-Geneva, Geneva University Hospitals, Switzerland; b University of Geneva, UNIGE, Geneva, Switzerland; c Division of Gynaecology, Department of Paediatrics, Gynaecology and Obstetrics, Geneva University Hospitals, Geneva, Switzerland; d University Centre of Legal Medicine Lausanne-Geneva, Geneva University Hospitals, Switzerland

**Keywords:** sexual assault, victims, PTSD, forensic medicine, intoxication

## Abstract

This study investigated whether total or partial amnesia following sexual assault increases the risk of developing PTSD symptoms. We retrospectively analyzed medical records of 209 adult patients consulting the Gynaecology Emergency Department at Geneva University Hospitals after alleged sexual assault (2015–2017). Data included presence of amnesia, age, victim-perpetrator relationship, intoxication, gynecological lesions, observed/reported PTSD symptoms, and psychiatric history. Amnesia was reported in 45.9% of cases, while 76.1% involved intoxication. PTSD-related symptoms (such as anxiety or dissociation) were noted in 37.3% of cases, with no significant difference between those with (32.3%) or without (41.6%) amnesia. Amnesia was more frequent in younger women, often intoxicated, and with unknown assailants. Our results indicate that amnesia after sexual assault is frequent but not associated with a higher prevalence of PTSD symptoms in the acute aftermath but is linked to other factors, such as age and intoxication.

## Introduction

### Sexual assault

Sexual victimisation is a major issue for society and public health (1). In Switzerland, one woman out of five (22%) has been a victim of sexual assault in the past, according to Amnesty International Switzerland (2). Sexual assault can lead to severe and frequent psychological post-traumatic consequences (3,4). In 2018, Guina et al. (5) showed that in an American population, sexual assaults and war combats were the traumatic events most likely to lead to psychotraumatic symptoms. Others have shown that half of sexual assault survivors meet criteria for PTSD three months after a sexual assault (6). These consequences could be characterised as post-traumatic stress disorder (PTSD), meaning that sexual assault meets the criteria for the diagnosis of PTSD according to the DSM-5 classification, or isolated PTSD symptoms such as anxiety, flashbacks, sleep problems, sadness and affect abrasion (7). Between 17% and 65% of sexual assault victims present with PTSD symptoms (3), some of them starting immediately after the assault. Amongst people suffering from PTSD in the US, sexual assault victims are one of the majority groups (8). Usually, symptoms tend to diminish with time (9,10). The presence and intensity of immediate and post-immediate symptoms are predictors of a future development of a characterised PTSD (11,12).

### Amnesia

Some victims of sexual assault report partial or total amnesia of the event, which means that they were unable to recall some moments or the entirety of the assault, but few studies directly address the topic. Jänisch et al. (13), in a German study from 2010, found almost 10% amnesia in a population of women consulting in a forensic department specialised in sexual assault victims. For most women this amnesia followed a psychotropic substance intake (including alcohol), taken willingly or unwillingly. LaHarpe et al. (14) in Geneva, Switzerland, showed that in a population of sexual assault victims consulting in forensic medicine between 2006 and 2012, 31% presented with partial or total amnesia of the assault, for most part under the effect of psychoactive substances taken willingly.

Juhascik et al., in 2007 (15), found that 43% of victims consulting in medical centres in the US were under the unwanted influence of drugs or alcohol during the sexual assault. In the same type of population in 2015, Xifró-Collsamata et al. (16,17) found that 30.7% of sexual assault victims were intoxicated during the assault and these victims tended to report amnesia more frequently than non-intoxicated victims. Intoxication is not the only mechanism leading to amnesia in sexual assault. Amnesia may also be the expression of a peritraumatic dissociative reaction (known as dissociative amnesia) which is understood as a psychological mechanism to cope with trauma and involves a disruption in the normal integration of consciousness and memory. This mechanism is conceptually and mechanistically different from alcohol-induced blackout, which is a neurobiological impairment of memory encoding and consolidation due to substance effects (5,18). Amnesia can also result from head trauma.

The impact of total or partial amnesia of a sexual assault victim on the future development of PTSD symptoms is not known yet. It is difficult to study as distinguishing the effects of the trauma from the eventual effects of substance intake is hard to assess. The studies on the subject are contradictory. Möller et al., in 2014 (19), found a protective effect of amnesia in the development of PTSD symptoms but Bryant et al., in 2009 (20), identified a short period of amnesia of the assault as a risk factor. Peritraumatic dissociative amnesia has been found to be a risk factor for developing PTSD (21). The absorption of benzodiazepines (the side effect of which is to induce amnesia), during or just after the assault, has been proven to be a risk factor for developing PTSD (22).

### Aim of the study

The aim of this study is to assess whether the presence of amnesia in female victims of sexual assault is associated with PTSD-related symptoms (rather than a formal diagnosis of PTSD, since the required duration criterion for making the diagnosis was not met in our cohort) at the time of consultation in the emergency department. We formed the hypothesis that amnesia is a risk factor for the development of PTSD symptoms such as deep anxiety, profound distress, avoidance or intrusion symptoms, intense physical or emotional reaction and dissociation. Another aim of this study was to describe the factors associated with amnesia, to better understand the population and circumstances under which amnesia usually occurs.

## Materials and methods

The population is women consulting in an emergency consultation for sexual victims in Geneva’s hospital, which takes place in the gynecological emergency unit any time of the day. The victims have access to a gynecological and a forensic consultation. The forensic physician describes the assault as told by the victim and reports the physical and psychiatric symptoms observed. The forensic doctor has no specific training on psychiatry and is a specialist in forensic medicine. They must ask the victim to recount the entire event and therefore document any difficulties or impossibilities in remembering part or all of the assault (partial or total amnesia).

The study was an observational descriptive retrospective study. It took place in the CURML (Centre Universitaire Romand de Médecine Légale) University Roman Forensic Medicine Centre. The data were extracted from the patient files of the gynecological and forensic reports for sexual assault, from 1 January 2015 to 31 December 2017. All victims were women aged 18 and older.

### Data collected

In total, 209 files were analysed. We recorded data on partial or total amnesia of the assault reported by the victim. For the purposes of this study, amnesia was defined as the inability to recall a continuous period of time related to the assault, rather than isolated details. For participants who reported complete amnesia for the event, the suspicion that they had been sexually assaulted was based on external evidence such as physical findings observed during the forensic examination, injuries, the presence of biological evidence, witness accounts or third-party information (for example, from police, friends, or relatives), or contextual cues discovered upon regaining consciousness. The following data were collected: the age of the victim, the link between victim and alleged assailant (known or unknown), the delay between the assault and the emergency consultation, the victim’s intake of alcohol or drugs, willingly or unwillingly (referred to as ‘intoxication of the victim’), the gynecological lesions observed during the examination, the PTSD symptoms (intense anxiety, dissociation, extreme sadness, suicidal thoughts, flashback) and the psychiatric history of the victim. PTSD symptoms were assessed according to the DSM-5 definition of PTSD: intrusion symptoms, avoidance, alteration in cognition, mood, arousal or reactivity, as observed by the forensic and gynaecologist physicians during the examination or self-reported by the patient and recorded by the examiners. Amnesia was excluded of these PTSD symptoms, even though ‘inability to recall key features of the trauma’ is listed as one of the symptoms of ‘negative thoughts or feelings’ in the DSM5 definition of PTSD. This exclusion was necessary for the purpose of this study because, without it, every subject suffering from amnesia would be considered as having PTSD symptoms, preventing us from comparing these variables. It should be noted that a full diagnostic interview for PTSD was not performed. Instead, the evaluation of PTSD symptoms was based on observation and patient self-report as recorded by the forensic physician and gynaecologist during the initial medical consultation, which typically occurred within days after the assault.

### Statistical analysis

Data were analysed using SPSS 25 software. Descriptive statistics together with bivariate analysis were conducted such as chi-square independence tests, followed by multivariate analysis such as logistic regressions. In this model, the dependent variable (DV) is the presence of PTSD symptoms. The independent variables (IV) are: amnesia, intoxication, gynecological lesions, and physical lesions as independent variables. Age, delay of consultation, link with the perpetrator, the type of aggression (rape or other), and psychiatric history were included as covariates. PTSD symptoms and amnesia were coded as dichotomous variables.

The significance threshold was *p* < 0.05.

## Results

### Population

During the inclusion period, 209 files were reviewed. Subjects were females between 18 and 71 years old and on average 30.1. Amongst them, 96 subjects (45.9%) suffered from partial or total amnesia of the assault. Ninety-six women knew their assailant and 113 (54.1%) did not. The mean delay between the assault and the consultation was 1.5 days, with a maximum of 30 days. Of the subjects, 87.6% consulted during the first three days after the assault.

Of the subjects, 159 (76.1%) were under the influence of drugs or alcohol during the assault. Seventy-eight patients (37.3%) presented with at least one PTSD symptom such as extreme sadness, intense anxiety and dissociative symptoms (confusion, agitation, lack of emotion, etc.). More than half of the study population had a psychiatric history (55%) such as an addiction (24.9%), depression (20.6%), an anxious disorder (9.1%), psychosis (7.7%), a bipolar disorder (3.3%) or a personality disorder (3.3%) (see Table 1).

**Table 1.** Population description.

**Variable**	***N***		**%**
Intoxication	159		76
Gynecological lesions	68		33
Psychiatric symptoms	78		37
Psychiatric history	115		55

### Bivariate analysis

PTSD symptoms were recorded in 32.3% of the subjects suffering from amnesia and in 41.6% of non-amnesic patients. Such difference was not significant (see Table 2).

**Table 2.** Cross table of amnesic subjects presenting with PTSD symptoms.

		**PTSD symptoms**		
		**No**	**Yes**	**Total**
**Amnesia**	**No**	66 (58.4%)	47 (41.6%)	113 (100.0%)
	**Yes**	65 (67.7%)	31 (32.3%)	96 (100.0%)
	**Total**	131 (62.7%)	78 (37.3%)	209 (100.0%)

Note: *p* > 0.05

Amnesic patients were significantly younger than non-amnesic (*p* < 0.05) with a mean age of 27.6 years old for amnesic subjects and 32.3 for non-amnesic.

Of non-amnesic women, 63.7% were assaulted by someone they knew. In contrast, 75% of amnesic women were assaulted by a stranger. Amnesic women were significantly (*p* < 0.001) more often assaulted by a stranger than non-amnesic women.

On average, amnesic subjects consulted after 1.0 days and non-amnesic after 2.0 days, this difference was not significant (*p* = 0.06).

Of non-amnesic subjects, 61.1% reported having taken drugs or alcohol against 93.8% of amnesic ones. This difference was significant (*p* < 0.001).

There was no difference between amnesic (32.3%) and non-amnesic subjects (32.7%) in gynecological lesions (*p* > 0.05).

There was no significant difference in psychiatric history among subjects presenting with or without amnesia, with 55.8% of non-amnesic women with a psychiatric history and 54.2% of amnesic women.

### Multivariate analysis

A logistic regression was conducted with PTSD symptoms as the dependent variable and amnesia, intoxication, gynaecological lesions, and physical lesions as independent variables. Age, delay of consultation, link with the perpetrator, type of aggression (rape or other), and psychiatric history were included as covariates. Collinearity was checked using a correlation matrix, which showed that all Pearson coefficients were less than 0.8, indicating that no variables were too correlated to be included in the model.

The model showed limited significance with a pseudo R^2^ of 0.065, meaning that 6.5% of the variance of the PTSD symptoms was explained by the independent variables. The delay of consultation, presence of gynaecological lesions, physical lesions, age, and psychiatric history were not significant, confirming the results of the bivariate analysis previously done. The type of aggression (rape or not) was the only significant predictor, with a beta coefficient of 1.3468 (p = 0.007), indicating that the presence of rape significantly increased the likelihood of PTSD symptoms. Intoxication, amnesia, link with the perpetrator, and other factors did not show a significant effect on PTSD symptoms in this model.

## Discussion

Partial or total amnesia after a sexual assault is not associated with the presence of PTSD symptoms during the emergency examination of the victim in our sample of 209 women. Amnesia was found to be associated with a younger age, willing or unwilling substance intake, and an unknown perpetrator. It was not possible in this retrospective study to discriminate whether amnesia was an effect of an intoxication or a dissociative amnesia, which is a consequence of a traumatic event, or both. These two different types of amnesia may have a different link to PTSD symptoms. The scientific literature shows indeed that peritraumatic dissociation (which could include dissociative amnesia) is a known risk factor for the development of PTSD symptoms and characterised PTSD (23). It is also possible that some victims did not want to talk about the assault and preferred to declare that they could not remember. We are also unable to estimate the amount and distinguish the types of substance that were taken.

In this study the average delay between assault and consultation was short. The longest delay was 30 days and the vast majority of the subjects consulted in the first three days after the assault. Thus the PTSD symptoms observed were of an acute reaction or even an immediate peritraumatic reaction. This cannot allow us to draw conclusions regarding more chronic symptoms. A prospective study with a re-evaluation after three and 12 months would allow us to evaluate the link between amnesia and more long-term post-traumatic symptoms.

This study showed a link between the type of aggression and the PTSD symptoms observed. Indeed, rape was a risk factor for developing PTSD symptoms. In Switzerland, rape has a specific definition which includes only the penetration of a penis in a vagina. The other types of aggression are called ‘sexual coercion’. Further studies should examine the link between the type of sexual aggression and its psychotraumatic consequences.

Half of the sample presented with amnesia (45.9%), which is high in comparison with other studies like the one conducted by LaHarpe et al. (14) on the same type of files but in an earlier period of time. This study found that 31% of the victims presented with amnesia in the consultations taking place between 2006 and 2012. The difference was that they also included victims under the age of 18 years old, with less alcohol and drug intake. Considering the frequency of amnesia, no matter the cause, health professionals working at emergency departments should be familiar with it and know that it can also be caused by trauma. Health professionals should know that victims might be unable to remember and refer to details that might be useful in deciding prophylactic treatment administration for sexually transmitted infections (STIs) or unwanted pregnancies. Gynaecologists and forensic physicians are confronted with amnesia of the victim during their consultations and must be informed of its frequency and link with intoxication, which should be searched for.

The population of this study was not representative of the general population of female victims of sexual assault because it was a selection of women who decided to consult and benefitted from a forensic examination. In this population, alleged offenders were for 51.4% unknown to the victims, which contrasts with what is usually found in victimisation studies in the general population (4). One might wonder if female victims of unknown perpetrators and presenting with amnesia might decide to consult more in an emergency department and in the short term, looking for a prescription of emergency contraception or STI prophylactic treatments or looking for evidence to file a complaint. In this study, victims suffering from amnesia tend to consult quicker than the other group. Another possibility is that women who remember the events may need a longer reflection time before asking for medical assistance.

This study has limits. All the evaluation was not made by a psychiatrist but by a forensic physician and a gynaecologist on call and without a standardised tool. Thus the evaluation of PTSD symptoms was dependent on the background of the physician and their interest in reporting psychiatric symptoms. Although there was a section in the files in which observations regarding psychiatric symptoms must be written down, this section may have been completed differently depending on the physician’s experience and background. Importantly, our study specifically evaluated PTSD symptoms, not the formal diagnosis of PTSD, as the clinical evaluations took place within days of the assault and did not meet the time criterion required for a diagnosis according to DSM-5. A prospective study involving a psychiatrist could be better to discriminate between PTSD symptoms linked to the assault or pre-existing psychiatric symptoms.

Several female patients are directly referred by the police when they come to file a complaint. Therefore, it was expected that the delay between the assault and the consultation could be long since some people may file a complaint several months or even years after the assault. Although some of these cases were indeed encountered, the average delay was less than 24 hours. That is a reason why ‘post-traumatic stress symptoms’ were searched for and not characterised PTSD. It seems that better training for first responders and better detection of at-risk individuals would allow for more targeted interventions. Indeed, the contact information for a psychotherapeutic consultation centre specialising in victim care was offered, and fewer than half of the consultees accepted it. A better awareness of forensic physicians and gynaecologists on psychiatric post-traumatic symptoms could help screen, identify and refer women in need of a specialised psychiatric consultation in the short and long term.

This study may also lack statistical power. A bigger population may have succeeded in discriminating between different symptoms and finding a link between each symptom and amnesia. It is indeed probable that flashbacks are less frequent when the victim presents with amnesia but the link with anxiety, dissociation and avoidance is unclear.

## Conclusion

In our sample, amnesia after a sexual assault was significantly more frequent in younger women, in case of drug and alcohol intake and when the assailant was unknown. Amnesia was not more frequently reported by women with PTSD symptoms at the moment of the emergency consultation after a sexual assault. The only predictor of PTSD found was the type of aggression (rape). A prospective study with a psychiatric evaluation after three and 12 months could provide a better evaluation of possible long- term PTSD symptoms associated with amnesia during and after a sexual assault.

## Availability of data and materials

The data that support the findings of this study are available from the corresponding author, CJ, upon reasonable request.
